# Family planning and multiple sclerosis – Part 1: Patient perspectives on treatment choices in Sweden

**DOI:** 10.1177/20552173261472661

**Published:** 2026-08-05

**Authors:** Alejandra Machado, Emma Pettersson, Fitsum Sebsibe Teni, Katharina Fink, Emilie Friberg

**Affiliations:** 1 Division of Insurance Medicine, Department of Clinical Neuroscience, 27106 Karolinska Institutet, Stockholm, Sweden; 2 Division of Neuro, Department of Clinical Neuroscience, 27106 Karolinska Institutet, Stockholm, Sweden

**Keywords:** Multiple sclerosis, disease-modifying therapies, family planning, insights, guidelines

## Abstract

**Background and Objectives:**

Family planning and treatment management in women with multiple sclerosis (MS) require complex decision-making. In most cases, patients express their intention to start a family, giving physicians time to monitor their MS and align treatment plans accordingly. However, not all pregnancies are planned, which can make timely communication with healthcare providers challenging. This study explores the dynamics between patients and healthcare professionals in MS care, focusing on family planning and treatment decisions before, during, and after pregnancy.

**Methods:**

People with MS aged 20–50 in the Swedish MS register (SMSreg) were invited to participate in an online survey in 2021. Responses of 3078 women were linked to SMSreg and other national register datasets. Descriptive statistics and multinomial logistic regression explored information sources, care, family planning discussion, treatment satisfaction, and its impact on their disease and breastfeeding decisions.

**Results:**

Women with MS reported receiving annual care from a neurologist (88%), nurse (68%) or general practitioner (46%), with satisfaction ranging from 40–77%. Most were treated with high efficacy (HE) disease-modifying treatments (DMTs) (66%), of whom 49% used rituximab. Treatment satisfaction was higher among younger women, milder EDSS, and those on HE DMTs. Women who became pregnant after their MS diagnosis (41%) were more likely to have discussed family planning with a physician (78%) compared to those who had not been pregnant post-diagnosis (59%).

**Discussion:**

In Sweden, women with MS generally have regular contact with healthcare professionals, though satisfaction varies. Family planning discussions were more frequent among women who became pregnant after diagnosis, highlighting the importance of proactive communication. Treatment choices influence both satisfaction and reproductive decisions, underscoring the need for clear guidance and shared decision-making. Real-world data also indicate that rituximab is commonly used during peripregnancy, likely due to its favourable safety profile and long-lasting effect.

## Introduction

Multiple sclerosis (MS) is commonly diagnosed among women of childbearing age thus, being diagnosed can affect considerations regarding family planning.^1^ Recent advancements in disease-modifying treatments (DMTs) have improved management,^2,3^ enabling women with MS to consider pregnancy contributing to an increase in birth rates.^4^ However, these therapies add complexity due to limited data on offspring safety during pregnancy and lactation.^2,5,6^ Pharmaceutical labels often advise against DMT use,^6,7^ yet real-world data suggest that some treatments may be safe when individual risk tolerance and clinical disease activity are considered.^8–10^ The risk/benefit balance must be considered at every stage of pregnancy.^6,11,12^ Hence, discussions with MS specialists are essential to ensure tailored and safe treatment options.

A key goal in MS treatment planning is to achieve disease stability 6 to 12 months before conception.^10,12^ Yet this is not always feasible, and unplanned pregnancies are common.^13^ While guidelines recommend discontinuing most of DMTs before conception, in practice, physicians may individualize treatment to reduce the risk of disease rebound during pregnancy.^14^ Moreover, pregnancy itself may reduce relapse rates, particularly in the second and third trimesters.^15,16^ However, postpartum relapses, are relatively common, with increased risk in the first three months postpartum.^15^ The extent of rebound varies based on prior disease activity, type of DMT, and breastfeeding choices.^16^ Yet, benefits of breastfeeding may outweigh the need to resume treatment immediately.^16^

Swedish guidelines for DMTs have categorized certain therapies according to pregnancy safety.^17,18^ Certain DMTs, such as teriflunomide and fingolimod, are contraindicated due to their potential teratogenicity.^2^ Natalizumab may carry a risk of rebound relapses if discontinued. Therefore, it is recommended to continue its use until late pregnancy or switch it to a high-efficacy, long-lasting drug before pregnancy.^6^ Contrary, glatiramer acetate and interferons, have been shown to be safe for use during pregnancy both for the mother and the fetus.^8–10^ Furthermore, off-label use of rituximab is common in Sweden,^19^ and the primary initial treatment choice given its affordability, safety and positive experiences reported in clinical practice.^20–23^ Furthermore, it has been suggested to have a long-lasting effect on MS disease activity after its suspension, which could protect against disease activity during pregnancy and the postpartum period.^24^ Therefore, it is often switched to when previous DMT is incompatible with pregnancy.^25,26^ Nonetheless, given the complexity and challenges of the timing, clinical status and potential risks, early discussions with MS specialists are crucial for a personalized approach.^3,6,27^

Healthcare professionals, including neurologists and nurses, along with online resources, are the primary sources of family planning information for people with MS (PwMS).^13,14^ A multicenter survey conducted in the USA and Europe indicated that, even though 81% of PwMS received information from healthcare providers, 56% reported that MS influenced their family planning, and many had not discussed this with their doctor.^14^ Furthermore, a Danish study, found nearly half of women with MS reported being uninformed about DMT use during pregnancy.^13^ This highlights the need for better patient education and structured discussions.

Overall, there is growing evidence that women with MS can safely conceive, breastfeed, and resume treatment according to their preference without significantly increasing the risk of relapse.^24^ However, there is a need for comprehensive evidence-based information to guide informed decisions about DMTs before, during and after pregnancy. This study aims to assess the frequency and nature of family planning discussions between women with MS and their healthcare providers, evaluate the patients’ insights and satisfaction with MS care, and evaluate the alignment of DMT choices with national guidelines (*see* Part 2 of this study).

## Materials and methods

An observational study was conducted based on a cross-sectional web-based survey of individuals with MS in Sweden linked to individual-level nationwide register data. The study was approved by the Swedish Ethical review Authority. Participants gave informed consent to participate in the study before taking part.

### Study population

Individuals aged 20–50 years who were alive, listed in the SMSreg, and living in Sweden were invited by Statistics Sweden to participate in a web-based (online) survey conducted between May and September 2021. Of those invited, 4412 (52%) answered the survey. The present study includes 3148 female respondents (71% of the total sample). Of these, 3078 women were included in the study based on their response to the question: ‘*Have you been pregnant during the time you have had MS?’* (response options: ‘No’, Yes, once’, or ‘Yes, more than once’, with the latter two combined as ‘Yes’). Accordingly, pregnancy groups were defined as: (1) ‘Pregnancy during MS’ *‒* women who became pregnant after MS diagnosis, and (2) ‘No pregnancy during MS’* ‒* women who did not become pregnant while living with MS.

### Data source

Using unique personal identity numbers, the survey data was linked to the following national register with a data cut-off date of 4 October 2021. This individual-level linkage using unique identifiers ensured that overlapping datasets and duplicate entries were avoided. The following registers were used: (1) Individual information was obtained from the Swedish MS register, which covers approximately 80% of all individuals with MS in Sweden since 2000 and collects data on disease onset, treatment, disease course, and disease severity. Disease severity was assessed with the most recently available Expanded Disability Status Scale (EDSS) score and cognitive processing speed assessed with the Symbol Digit Modalities Test (SDMT); (2) The Longitudinal Integrated Database for Health Insurance and Labour Market Studies (LISA), held by Statistics Sweden, was used to obtain socio-demographic variables as of 31 December 2019 (sex, educational level, country of birth, marital status, living with children at home, and type of living area).

Age was calculated as the difference between the date of birth (extracted from the SMSreg) and the date of survey response.

### Outcomes

Responses on eleven specific close-ended questions related to family planning, pregnancy, information sources and support from healthcare professionals as well as treatment information, its impact and satisfaction were included (see Annex in Supplementary material for further details).

Clinical data from SMSreg included time since onset, time since diagnosis, type of MS (relapsing-remitting, secondary-progressive, primary-progressive, missing) and MS severity, with the most recent EDSS. Scores were set to missing if the assessment was more than three years prior the survey response date (*n =* 347) or if the score was recorded as 0 but the participant had progressive MS (*n =* 5).^28^ EDSS scores were categorized as mild (EDSS=0–2.5), moderate (EDSS=3–5.5) or severe disability (EDSS=6–9.5). Nearly all (3030, 98.4%) of the women had valid data on disease-modifying therapy (DMT), with treatment start and/or stop dates. The latest treatment was either the ongoing treatment at the time of the data extraction (4 October 2021) or the latest treatment registered in the previous three years. No treatment status was set for cases with no therapy registered within this period. Only DMTs were considered for this study, excluding all treatments for MS symptomatology. DMTs were also classified as high-efficacy and non-high-efficacy, following the categorization used in one of our previous studies^29^ (see details on caption of Table 1).

**Table 1.** Cohort demographics and clinical characteristics from register data.

	No pregnancy during MS (*N =* 1803)	Pregnancy during MS (*N =* 1275)	Overall (*N =* 3 078)	*p*-value^a^
Age groups, *n* (%)				.**000**
20-29 years	236 (13.1%)	51 (4.0%)	287 (9.3%)	
30–39 years	524 (29.1%)	462 (36.2%)	986 (32.0%)	
40–49 years	842 (46.7%)	689 (54.0%)	1531 (49.7%)	
50 + years	201 (11.2%)	73 (5.7%)	274 (8.9%)	
Born in Sweden, *n* (%)				.757
Yes	1590 (88.2%)	1129 (88.6%)	2719 (88.3%)	
No	213 (11.8%)	146 (11.5%)	359 (11.7%)	
Type of living area based on municipality density, *n* (%)				.952
City	789 (43.8%)	551 (43.2%)	1340 (43.5%)	
Town/suburb	701 (38.9%)	502 (39.4%)	1203 (39.1%)	
Rural area	313 (17.4%)	222 (17.4%)	535 (17.2%)	
Educational level, *n* (%)				.**000**
Primary	84 (4.7%)	38 (3.0%)	122 (4.0%)	
High school	630 (34.9%)	348 (27.3%)	978 (31.8%)	
University/college	1089 (60.4%)	889 (69.7%)	1978 (64.3%)	
Married/cohabitant, *n* (%)				.**000**
No	1124 (62.3%)	577 (45.3%)	1701 (55.3%)	
Yes	679 (37.7%)	698 (54.8%)	1377 (44.7%)	
Children under age 20, *n* (%)				.**000**
No	995 (55.3%)	225 (17.7%)	1220 (39.7%)	
Yes	808 (44.7%)	1050 (82.3%)	1858 (60.3%)	
Type of MS, *n* (%)				.**009**
Relapsing-remitting MS (RRMS)	1679 (93.6%)	1158 (91.9%)	2837 (92.9%)	
Primary progressive MS (PPMS)	32 (1.8%)	14 (1.1%)	46 (1.5%)	
Secondary progressive-MS (SPMS)	84 (4.7%)	88 (7.0%)	172 (5.6%)	
Unknown	8	15	23	
EDSS, *n* (%)				.157
Mild	1149 (63.7%)	816 (79.1%)	1965 (80.7%)	
Moderate	198 (11.9%)	163 (15.8%)	361 (14.8%)	
Severe	55 (3.1%)	53 (5.1%)	108 (4.4%)	
Unknown	401	243	644	
Time since diagnosis (years)				.**000**
Mean (SD)	7.1 (5.8)	11.8 (6.4)	9.1 (6.5)	
Median (Min, Max)	6.0 (2.0, 10.0)	12.0 (7.0, 16.3)	8.0 (4.0, 13.0)	
Unknown	123	75	198	
Time since onset (years)				.**000**
Mean (SD)	8.9 (6.7)	14.3 (6.8)	11.2 (7.3)	
Median (Min, Max)	8.0 (4.0, 13.0)	14.0 (9.0, 19.0)	10.0 (5.0, 16.0)	
Unknown	137	72	209	
Latest DMT, *n* (%)				.**000**
No treatment	99 (5.5%)	128 (10.0%)	227 (7.4%)	
Alemtuzumab	15 (0.8%)	15 (1.2%)	30 (1.0%)	
Cladribine	31 (1.7%)	21 (1.7%)	52 (1.7%)	
Dimethyl fumarate	215 (11.9%)	119 (9.3%)	334 (10.9%)	
Fingolimod	85 (4.7%)	74 (5.8%)	159 (5.2%)	
Glatiramer acetate	44 (2.4%)	28 (2.2%)	72 (2.3%)	
HSCT	29 (1.6%)	29 (2.3%)	58 (1.9%)	
Interferon	81 (4.5%)	63 (4.9%)	144 (4.7%)	
Natalizumab	221 (12.3%)	178 (14.0%)	399 (13.0%)	
Ocrelizumab	16 (0.9%)	10 (0.8%)	26 (0.8%)	
Ofatumumab	<5 (0.1%)	0 (0.0%)	<5 (0.0%)	
Rituximab	922 (51.1%)	585 (45.9%)	507 (49.0%)	
Teriflunomide	44 (2.4%)	25 (2.0%)	69 (2.2%)	
Latest DMT classification^b^, *n* (%)				.**000**
No treatment	99 (5.5%)	128 (10.0%)	227 (7.4%)	
High efficacy	1204 (66.8%)	817 (64.1%)	2021 (65.6%)	
Non-high efficacy	500 (27.7%)	330 (25.9%)	830 (27.0%)	

Note: Cell sizes less than 5 are suppressed as per data access and privacy requirements.

a Pearson's chi-squared test; Fisher's exact test; Wilcoxon rank sum test; A *p*-value of < .05 was considered as statistically significant (marked in bold).

b High efficacy DMTs: alemtuzumab, hematopoietic stem cell transplantation (HSCT), natalizumab, ocrelizumab, ofatumumab, and rituximab. Non-high-efficacy DMTs: cladribine, dimethyl fumarate, fingolimod, glatiramer acetate, interferons (interferon beta-1a, interferon beta-1b, and peginterferon beta-1a), and teriflunomide.

Abbreviations: MS: multiple sclerosis; EDSS: expanded disability status scale; DMT: disease-modifying treatment.

### Statistical analysis

Descriptive statistics were used to summarize the demographic and clinical characteristics of the sample, as well as questionnaire responses. Differences in the distribution of these characteristics across MS pregnancy groups were examined with parametric or non-parametric tests, where appropriate.

Multinomial logistic regression was used to estimate the association between covariates and treatment satisfaction among women with MS. A single fully adjusted model was fitted, with covariates selected based on clinical relevance, prior literature, and variables showing differences in preceding chi-squared analyses, primarily reflecting clinical characteristics and age. Results are presented in odds ratios (OR) with a 95% confidence interval (CI). All results were deemed significant at a p-value threshold of <.05. Analyses were performed using R software (version 4.5.0).

## Results

### Participants characteristics

The total sample consisted of 3078 women, of whom 41% (*n =* 1275) reported being pregnant at least once while having MS (hereafter referred to as ‘pregnancy during MS’; Table 1), of which 61 were pregnant at the time the survey was conducted (Table S1). The remaining 59% who had not been pregnant while having MS, were either younger (<30 years) or older (>50 years) than the ‘pregnancy during MS’ group, had proportionally lower education level, were more often single, and had fewer children under 20 years of age living at home. In the ‘pregnancy during MS’ group, women had more often converted to secondary progressive MS, had a longer average disease duration, and were more often untreated (Table 1).

Overall, only 7.4% of women were untreated, while the majority received a DMT: 65.6% were on high efficacy (HE) therapies and 27.0% on non-high-efficacy treatments. Among those treated, nearly half were prescribed with rituximab (49%), followed by natalizumab (13%) and dimethyl fumarate (10.9%; Table 1).

### Sources of information and support

In survey questions about sources of information or support, women with MS reported receiving care primarily from their neurologist, either a few times per year (31%) or at least once in the past year (55%). They also had frequent contact with their general practitioner (GP; 25%), a nurse (25%) or a physician at another specialist clinic (16%) during the past year. Nurses were the health professionals most frequently contacted monthly (11%; see Table S2).

Overall, women reported receiving support to a greater extent from physicians at a neurology clinic (77%), nurses (60%) and GPs (40%). Many respondents expressed their wish for additional support from physiotherapists (18%), neurologists (17%), psychologists (17%), neurological rehabilitation services (16%), or chiropractors (14%) (Table S3). For MS treatment information (see Table S4, Q2), the main source were physicians (64%), the internet (19%), and other healthcare professionals (12%). The remaining 5% turned to patient associations, personal contacts, or other sources. These patterns did not differ between women who experienced pregnancy during their MS and those who had not (*p = .*231; Table S4).

### Treatment satisfaction

When compared to women satisfied with their treatment, women reporting ‘somewhat satisfied’ or ‘not satisfied’, were less likely to be on HE (OR: 0.62, 95% CI: 0.42–0.92; and OR: 0.14, 95% CI: 0.08–0.25, respectively) or on non-high efficacy DMTs (‘not satisfied’: OR: 0.23, 95% CI: 0.12–0.46) (Table 2) relative to the no-treatment group (reference category). They also had higher disability levels (moderate and severe EDSS), particularly women not satisfied with their treatment (OR: 5.07, 95% CI: 2.61–9.83 and OR: 4.17, 95% CI: 2.58–6.75, respectively). Undergoing pregnancy during MS or time since onset did not impact treatment satisfaction. Descriptives stratified by groups confirmed the above findings. However, compared to women with no pregnancy during MS, those pregnant during MS and not satisfied with their treatment had slightly higher percentage of no DMTs, milder EDSS and were between 40–49 years of age (Table S5).

**Table 2.** Multinomial logistic regression for treatment satisfaction.

	Somewhat			No		
	AOR^a^	95% CI	*p*-value	AOR^a^	95% CI	*p*-value
*Pregnancy*						
No pregnancy during MS	ref.			ref.		
Pregnancy during MS	1.02	0.82–1.26	.881	0.77	0.46–1.28	.313
*Latest DMT*						
No treatment	ref.			ref.		
High efficacy	0.62	0.42–0.92	**.017**	0.14	0.08–0.25	**<.001**
Non high efficacy	1.18	0.78–1.76	.433	0.23	0.12–0.46	**<.001**
*EDSS*						
Mild	ref.			ref.		
Moderate	2.24	1.69–2.97	**<.001**	5.07	2.61–9.83	**<.001**
Severe	4.17	2.58–6.75	**<.001**	20.3	9.34–44.1	**<.001**
Missing	1.40	1.09–1.79	**.008**	2.56	1.37–4.79	**.003**
*Age groups*						
20–29 years	ref.			ref.		
30–39 years	1.55	1.03–2.34	**.038**	1.00	0.36–2.79	.997
40–49 years	1.39	0.92–2.11	.122	0.73	0.26–2.06	.550
50–59 years	1.25	0.74–2.11	.399	0.51	0.14–1.80	.292
Time since onset (years)	0.99	0.98–1.01	.337	1.02	0.99–1.06	.235

Note: Reference category for the outcome = yes (satisfied with treatment). Reference level for each categorical predictor (ref.). A single fully adjusted model was fitted. A *p*-value of < .05 was considered as statistically significant (marked in bold). Covariates were selected based on clinical relevance, prior literature, and variables showing differences in preceding descriptive analyses. Because several variables captured overlapping aspects of disease progression (e.g., MS type, EDSS, time since onset, time since diagnosis), multicollinearity was assessed and only EDSS and time since onset were retained in the final model based on clinical interpretability and statistical performance.

Abbreviations: AOR: adjusted odds ratio; CI: confidence interval; DMT: disease modifying treatment; EDSS: expanded disability status scale.

### Information on family planning and impact of MS before and after pregnancy

Concerning family planning, nearly half of the surveyed women (43%) reported having discussed it with their neurologist, while 22% had not, and 35% selected ‘not applicable’ (Table S4, Q1). However, responses varied significantly between groups (*p = .*015). Among women who experienced pregnancy during their MS, 74% had discussed family planning with their neurologist. In contrast, women who had not been pregnant while having MS, mostly responded ‘not applicable' (51%) or stated they had not discussed it (28%). As a follow up question, women who experienced pregnancy during their MS were asked: ‘*Overall, how has your pregnancy/pregnancies affected your MS?’* More than half (56%) responded ‘neither positively nor negatively', while 23% reported a positive and 19% a negative impact (Table S4, Q3). Furthermore, when asked whether treatment had influenced their decision to breastfeed, responses were evenly distributed: 27% said ‘no’, 28% chosen ‘not to breastfeed due to MS treatments’, and 31% postponed treatment to breastfeed. A small percentage (6%) reported not breastfeeding for other reasons (Table S4, Q4).

### Treatment and pregnancy

**Figure 1.**
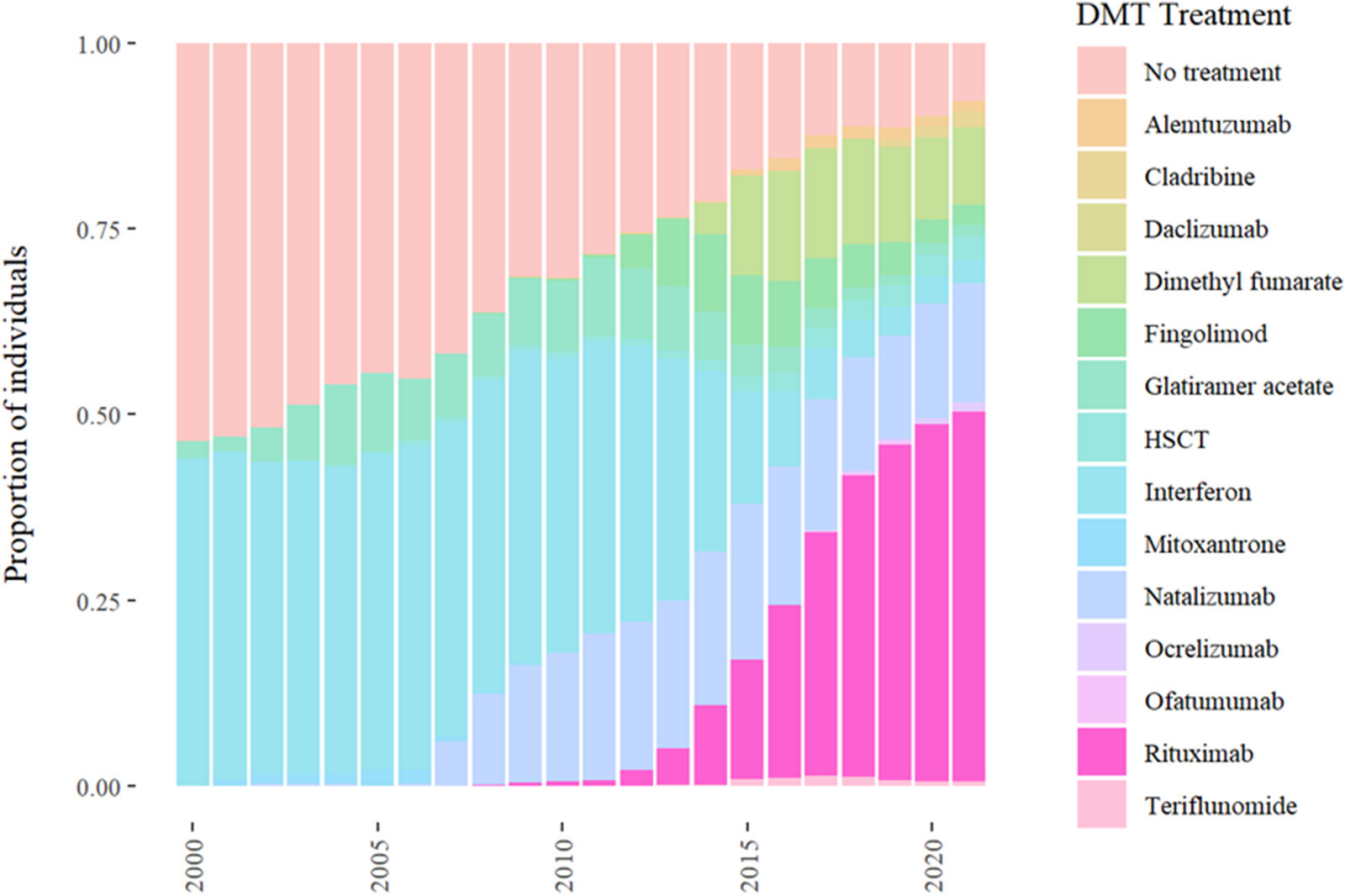
Distribution of DMT treatments by calendar year among respondents of childbearing ages (20–40 years). This subset (*n =* 2524) includes individuals followed from the date of MS diagnosis until the end of the study period (October 4, 2021), contributing data during periods when they were aged 20–40 years.

Note: In case of more than one treatment in a single year, the treatment used during the largest portion of the year is shown. HSCT: hematopoietic stem cell transplantation.

We further analyzed a subset of 2542 women of childbearing age (20–40 years) from the original study population, examining their treatment patterns using registry data from diagnosis through the end of the study period (October 2021). Figure 1 illustrates the shift in DMT use over time. In the early 2000s, more than half were either untreated or on first-generation therapies (interferons or glatiramer acetate). Over the following decade, the proportion of untreated women declined, while second generation DMTs – introduced around 2010 (e.g., fingolimod, dimethyl fumarate, etc.), became more commonly used. The introduction of rituximab as off-label treatment for MS began in 2008 and gradually increased to take over as the favoured treatment by 2014. By 2021, rituximab accounted for half of all DMT prescriptions among women of childbearing age in Sweden.

## Discussion

In this cross-sectional study of Swedish women with MS aged between 20 and 50, just under half (41%) reported being pregnant while diagnosed with MS, a proportion slightly higher than the estimates suggesting that one in three women become pregnant following MS onset or diagnosis.^30,31^ Women who experienced pregnancy during their MS tended to have a longer disease duration and were more likely to have converted to a progressive MS or be untreated at the time of the survey. These patterns suggest they may have developed MS at a younger age, which can affect how pregnancy relates to disability.^32^

Overall, women with MS reported that care, treatment and support – at least once per year – were primarily received from physicians at the neurological clinics, nurses and GPs, highlighting the reliance on both specialist and primary healthcare services. Most women with MS were satisfied with the support received; however, 17% reported unmet need for additional support from other specialists such as physiotherapists, psychologists, and neurological rehabilitation services. Women with lower levels of MS-related disability reported a greater need for psychological support, which may highlight the significant impact of fatigue and other invisible symptoms and their role in prompting individuals to seek help in managing MS.^33^ These findings converge with previous evidence of showing extensive healthcare utilization in MS and support the need for multidisciplinary care models.^34^

The relatively low frequency of information provision by neurologists observed in our study may be influenced by several factors, including clinical routines, time constraints, and the structure of follow-up visits. For example, patients receiving infusion therapies, as well as those with milder disease, may have less frequent consultations, which could partly explain this finding. Variability in patient satisfaction may also reflect differences in individual expectations and perceived adequacy of the information provided, where some women may find annual communication sufficient, while others may prefer more frequent or detailed information.

Family planning discussions with a neurologist were more common among women who became pregnant after MS diagnosis. However, a considerable proportion of women – both those who became pregnant (13%) and those who did not (28%) – had not engaged in such discussions. These findings were not surprising considering unplanned pregnancies are not uncommon.^12,13^ Previous research from Switzerland found that pregnancy is not consistently addressed in clinical encounters.^27^ In that study only, 17% of women with MS reported that their neurologist initiated discussions about pregnancy-related discussions at each consultation, while 26% indicated that the topic was never raised – findings that are consistent with our study.^27^ Together, these findings suggest that, despite the importance of family planning in MS management, many women with MS may not receive sufficient counselling on family planning.^13^ This may help explain to the reliance on online information resources and indicates a need to strengthen communication in clinical care.^27^

Furthermore, among women who experienced pregnancy during their MS, the impact of pregnancy was most commonly reported as neutral (i.e., neither positive nor negative). A follow-up open question (data not shown) suggested a range of experiences, from no perceived impact (e.g., ‘*no impact at all’*) to concerns about worsening disease (e.g., ‘*no big difference’*, ‘*no worsening in their MS but constant fear or worry’*) or variability between pregnancies (e.g., ‘*different impact depending on whether it was their first, second, or third pregnancy’*). Positive experiences were often described as improved well-being during pregnancy (e.g., ‘*feeling good’*), while negative experiences were typically associated with increased disease activity postpartum. Breastfeeding choices varied, with women reporting either postponing their treatment, avoiding breastfeeding due to treatment or no impact of treatment on their decision. These findings highlight the variability of experiences and the importance of individualized counselling. The high rates of breastfeeding may also reflect strong public health recommendations in Sweden, although guidance for women with MS remains treatment-dependent.^16^

Overall, two-thirds of the surveyed women were on high efficacy DMT, of which rituximab accounted for almost half, reflecting its widespread use in Sweden, even among women of childbearing age.^19,23,24^ This contrasts sharply with practices in other European settings, where prior to conception, women of childbearing age were more often treated with non-HE therapies (53–64%) than with HE therapies (23–24%).^5,35^ These differences may reflect variations in national prescribing practices and evolving perceptions of DMT use around pregnancy, as concerns regarding future pregnancy have been associated with lower exposure to DMTs among women with MS.^36^ Only a few women remained untreated, typically those with greater disability and progressive forms of MS,^29,37^ for whom treatment options may be limited.^29^ Women most frequently reported receiving information about MS treatments from their physician, while other healthcare professionals and the online resources were also identified as important. These findings align with previous studies from the past decade,^13,38^ highlighting the broader implications for how health-related information is communicated and accessed.^38^ Overall, 77% of respondents reported being satisfied with their treatment, with satisfaction particularly high among those receiving high-efficacy DMTs (69.1%). In comparison, 18% reported being somewhat satisfied, 3% were not satisfied, and 2% did not respond. However, compared to those who reported being satisfied with their treatment, respondents who answered ‘somewhat satisfied’ or ‘not satisfied’ were less likely to be on high-efficacy treatments, and more likely to have moderate to severe EDSS scores. Furthermore, there were no differences in treatment satisfaction between women who experienced pregnancy during their MS and those who had not. Our findings add a different angle to those of Vestergaard et al.,^13^ who showed that many patients lack clear information about DMT-related reproductive risks. While their study highlights informational gaps in patient awareness, our results suggest that treatment satisfaction is more closely tied to treatment efficacy and disability level, rather than to pregnancy history. Nevertheless, perceptions may vary depending on individual expectations, disease status, and the perceived adequacy of the information provided during these encounters. Together, the two perspectives point to the importance of both good communication and effective therapy in shaping patients’ experiences with MS treatment.

An additional aspect to consider is therapeutic inertia, defined as the failure to initiate or escalate treatment despite evidence of suboptimal disease control. In MS, this may involve continuing lower-efficacy therapies or delaying treatment escalation. Although not directly assessed, the high use of high-efficacy therapies may indicate a relatively low degree of therapeutic inertia in Sweden.^36^ However, the presence of untreated patients and those reporting lower satisfaction may still reflect delayed treatment optimization.

These findings also highlight the importance of patient empowerment in MS care, particularly in the context of reproductive decision-making. Structured preconception counselling, educational programs, and decision-support tools can support informed choices regarding treatment, pregnancy, and breastfeeding. Strengthening shared decision-making is essential, as patients must balance disease control with reproductive goals, and may also contribute to improved satisfaction and treatment outcomes. Despite the strengths of combining survey data with national registers, some limitations should be mentioned. The survey format limits the ability to explore the reasoning behind participant's responses, which may affect the generalizability of the findings. For example, the survey did not include follow-up questions to capture the underlying reasons why some participants did not receive family planning counselling, limiting our ability to interpret this finding in greater depth. Furthermore, the cross-sectional design restricts causal interpretations, and self-reported data may be subject to recall or reporting bias. Responses may also be influenced by individual expectations and current clinical status, which are inherent considerations in the interpretation of these findings. Moreover, those who chose to respond to the survey may differ systematically from non-respondents, potentially limiting generalizability. Additionally, important contextual factors such as socioeconomic status, cultural background, or access to specialized care were not fully captured, which may influence treatment decisions and reproductive outcomes. Future studies should aim to explore these reproductive and care-related experiences longitudinally and further examine how tailored counselling and treatment strategies can better support women with MS during key life stages such as pregnancy and early motherhood.

In conclusion, managing MS in women of childbearing age requires a nuanced, individualized approach that carefully balances maternal health with fetal safety. The increasing use of high-efficacy therapies and, induction therapies with long-lasting effects, combined with greater opportunities for pregnancy in women with MS, underscores the need for timely, informed treatment planning to prevent disease activity, functional decline and fetal risk. This real-world study offers valuable insights into how Swedish women with MS receive information and support regarding family planning and treatment options, emphasizing the importance of ongoing patient-centered dialogue throughout all stages of reproductive care. Strengthening communication between patients and providers, particularly during preconception and postpartum periods, is essential for optimizing outcomes. Further research and up-to-date clinical guidelines are needed to support clinicians and women with MS in making well-informed decisions within increasingly complex therapeutic landscape.

## Supplemental Material

sj-docx-1-mso-10.1177_20552173261472661 - Supplemental material for Family planning and multiple sclerosis – Part 1: Patient perspectives on treatment choices in Sweden Supplemental material, sj-docx-1-mso-10.1177_20552173261472661 for Family planning and multiple sclerosis – Part 1: Patient perspectives on treatment choices in Sweden by Alejandra Machado, Emma Pettersson, Fitsum Sebsibe Teni, Katharina Fink and Emilie Friberg in Multiple Sclerosis Journal – Experimental, Translational and Clinical

sj-docx-2-mso-10.1177_20552173261472661 - Supplemental material for Family planning and multiple sclerosis – Part 1: Patient perspectives on treatment choices in Sweden Supplemental material, sj-docx-2-mso-10.1177_20552173261472661 for Family planning and multiple sclerosis – Part 1: Patient perspectives on treatment choices in Sweden by Alejandra Machado, Emma Pettersson, Fitsum Sebsibe Teni, Katharina Fink and Emilie Friberg in Multiple Sclerosis Journal – Experimental, Translational and Clinical
