# Supplementary material for: Family planning and multiple sclerosis – Part 1: Patient perspectives on treatment choices in Sweden
Source: Mult Scler J Exp Transl Clin. 2026 Aug 5;12(3):20552173261472661. doi: 10.1177/20552173261472661 (PMC13443254; doi:10.1177/20552173261472661)
Supplement: Supplementary file 1 [file sj-docx-1-mso-10.1177_20552173261472661.docx]

**SUPPLEMENTARY** **MATERIAL**

*Survey questions related to pregnancy and children*

**Table S1. Frequency and proportions (%) of responses, considering whether the respondent is/was pregnant or not while having MS and overall**

|  | No pregnancy during MS | Pregnancy during MS | Overall | P-value |
| --- | --- | --- | --- | --- |
|  | (N=1803) | (N=1275) | (N=3078) |  |
| *Have you been pregnant during the time you have had MS?* *** | | | | ***-*** |
| Yes, once | 0 (0%) | 639 (50.1%) | 639 (20.8%) |  |
| Yes, several times | 0 (0%) | 636 (49.9%) | 636 (20.7%) |  |
| No | 1803 (100%) | 0 (0%) | 1803 (58.6%) |  |
| *Are you pregnant now?* | |  |  | **-** |
| Yes | 0 (0%) | 61 (4.8%) | 61 (2.0%) |  |
| No | 0 (0%) | 1207 (94.7%) | 1207 (39.2%) |  |
| Missing | 1803 (100%) | 7 (0.5%) | 1810 (58.8%) |  |
| *Do you have children of your own?* | | | | **<.001** |
| Yes | 910 (50.5%) | 1178 (92.4%) | 2088 (67.8%) |  |
| No | 892 (49.5%) | 97 (7.6%) | 989 (32.1%) |  |
| Missing | 1 (0.1%) | 0 (0%) | 1 (0.0%) |  |
| *Who do you live with? *** | | | |  |
| Children | 789 (43.8) | 1070 (83.9%) | 1859(60.4%) |  |

**This question was used to stratify the sample as having been pregnant or not while having MS.*

*** Potential alternatives were: ‘No one’, ‘Husband/wife/partner’, ‘Parents or siblings’, and ‘Other adults*

*Survey questions related to information source*

**Table S2*.* Frequency and proportions (%) of all women’s (pregnant or not pregnant while having MS) responses to the question: *How often do you receive care/treatment/support from the following professionals? Including both physical visits and, for example, telephone contacts***

| ***Healthcare professional*** | ***All participants***  ***or subgroups*** | ***N,n*** | ***Every week*** | ***Every month*** | ***A few times a year*** | ***The past year*** | ***The past 2-3 years*** | ***More seldom/ never*** |
| --- | --- | --- | --- | --- | --- | --- | --- | --- |
| **Physician at a neurology clinic** | All | 3058 | 3 (0.1) | 62 (2.0) | 945 (30.9) | 1693 (55.4) | 234 (7.7) | 121 (4.0) |
|  | No pregnancy during MS | 1793 | 2 (.1) | 44 (2.5) | 595 (33.2) | 959 (53.5) | 120 (6.7) | 73 (4.1) |
|  | Pregnancy during MS | 1265 | 1 (.1) | 18 (1.4) | 350 (27.7) | 734 (58.0) | 114 (9.0) | 48 (3.8) |
| **Neurological rehabilitation** | All | 3007 | 20 (0.7) | 50 (1.7) | 136 (4.5) | 251 (8.3) | 265 (8.8) | 2285 (76.0) |
|  | No pregnancy during MS | 1763 | 13 (.7) | 34 (1.9) | 83 (4.7) | 174 (9.9) | 146 (8.3) | 1313 (74.5) |
|  | Pregnancy during MS | 1244 | 7 (.6) | 16 (1.3) | 53 (4.3) | 77 (6.2) | 119 (9.6) | 972 (78.1) |
| **Physician at another specialist clinic** | All | 3015 | 7 (0.2) | 30 (1.0) | 245 (8.1) | 476 (15.8) | 272 (9.0) | 1985 (65.8) |
|  | No pregnancy during MS | 1762 | 5 (.3) | 21 (1.2) | 146 (8.3) | 264 (15.0) | 153 (8.7) | 1173 (66.6) |
|  | Pregnancy during MS | 1253 | 2 (.2) | 9 (.7) | 99 (7.9) | 212 (16.9) | 119 (9.5) | 812 (64.8) |
| **General practitioner at a primary healthcare center** | All | 3030 | 3 (0.1) | 36 (1.2) | 595 (19.6) | 751 (24.8) | 452 (14.9) | 1193 (39.4) |
|  | No pregnancy during MS | 1771 | 2 (.1) | 19 (1.1) | 355 (20.0) | 426 (24.1) | 264 (14.9) | 705 (39.8) |
|  | Pregnancy during MS | 1259 | 1 (.1) | 17 (1.4) | 240 (19.1) | 325 (25.8) | 188 (14.9) | 488 (38.8) |
| **Nurse** | All | 3035 | 17 (0.6) | 324 (10.7) | 934 (30.8) | 772 (25.4) | 185 (6.1) | 803 (26.5) |
|  | No pregnancy during MS | 1775 | 10 (.6) | 194 (10.9) | 605 (34.1) | 437 (24.6) | 101 (5.7) | 428 (24.1) |
|  | Pregnancy during MS | 1260 | 7 (.6) | 130 (10.3) | 329 (26.1) | 335 (26.6) | 84 (6.7) | 375 (29.8) |
| **Medical social worker** | All | 3016 | 20 (0.7) | 81 (2.7) | 91 (3.0) | 190 (6.3) | 266 (8.8) | 2368 (78.5) |
|  | No pregnancy during MS | 1766 | 14 (.8) | 56 (3.2) | 68 (3.9) | 118 (6.7) | 164 (9.3) | 1346 (76.2) |
|  | Pregnancy during MS | 1250 | 6 (.5) | 25 (2.0) | 23 (1.8) | 72 (5.8) | 102 (8.2) | 1022 (81.8) |
| **Psychologist** | All | 3021 | 35 (1.2) | 78 (2.6) | 70 (2.3) | 153 (5.1) | 208 (6.9) | 2477 (82.0) |
|  | No pregnancy during MS | 1764 | 23 (1.3) | 46 (2.6) | 42 (2.4) | 97 (5.5) | 113 (6.4) | 1443 (81.8) |
|  | Pregnancy during MS | 1257 | 12 (1.0) | 32 (2.5) | 28 (2.2) | 56 (4.5) | 95 (7.6) | 1034 (82.3) |
| **Psychotherapist** | All | 3004 | 13 (0.4) | 25 (0.8) | 42 (1.4) | 54 (1.8) | 96 (3.2) | 2774 (92.3) |
|  | No pregnancy during MS | 1753 | 9 (.5) | 13 (.7) | 29 (1.7) | 31 (1.8) | 60 (3.4) | 1611 (91.9) |
|  | Pregnancy during MS | 1251 | 4 (.3) | 12 (1.0) | 13 (1.0) | 23 (1.8) | 36 (2.9) | 1163 (93.0) |
| **Occupational therapist** | All | 3014 | 18 (0.6) | 38 (1.3) | 101 (3.4) | 297 (9.9) | 281 (9.3) | 2279 (75.6) |
|  | No pregnancy during MS | 1768 | 13 (.7) | 26 (1.5) | 58 (3.3) | 197 (11.1) | 181 (10.2) | 1293 (73.1) |
|  | Pregnancy during MS | 1246 | 5 (.4) | 12 (1.0) | 43 (3.5) | 100 (8.0) | 100 (8.0) | 986 (79.1) |
| **Physiotherapist** | All | 3030 | 83 (2.7) | 89 (2.9) | 196 (6.5) | 421 (13.9) | 382 (12.6) | 1859 (61.4) |
|  | No pregnancy during MS | 1772 | 56 (3.2) | 53 (3.0) | 123 (6.9) | 257 (14.5) | 216 (12.2) | 1067 (60.2) |
|  | Pregnancy during MS | 1258 | 27 (2.1) | 36 (2.9) | 73 (5.8) | 164 (13.0) | 166 (13.2) | 792 (63.0) |
| **Speech therapist** | All | 3007 | 3 (0.1) | 4 (0.1) | 9 (0.3) | 32 (1.1) | 63 (2.1) | 2896 (96.3) |
|  | No pregnancy during MS | 1755 | 1 (.1) | 4 (.2) | 7 (.4) | 27 (1.5) | 37 (2.1) | 1679 (95.7) |
|  | Pregnancy during MS | 1252 | 2 (.2) | 0 (0) | 2 (.2) | 5 (.4) | 26 (2.1) | 1217 (97.2) |
| **Naprapathy / chiropractor** | All | 3013 | 10 (0.3) | 57 (1.9) | 168 (5.6) | 157 (5.2) | 177 (5.9) | 2444 (81.1) |
|  | No pregnancy during MS | 1761 | 7 (.4) | 24 (1.4) | 88 (5.0) | 97 (5.5) | 96 (5.5) | 1449 (82.3) |
|  | Pregnancy during MS | 1252 | 3 (.2) | 33 (2.6) | 80 (6.4) | 60 (4.8) | 81 (6.5) | 995 (79.5) |
| **Rehabilitation coordinator** | All | 3006 | 3 (0.1) | 8 (0.3) | 27 (0.9) | 88 (2.9) | 103 (3.4) | 2777 (92.4) |
|  | No pregnancy during MS | 1756 | 2 (.1) | 6 (.3) | 17 (1.0) | 57 (3.2) | 67 (3.8) | 1607 (91.5) |
|  | Pregnancy during MS | 1250 | 1 (.1) | 2 (.2) | 10 (.8) | 31 (2.5) | 36 (2.9) | 1170 (93.6) |
| **Dietician** | All | 3009 | 3 (0.1) | 11 (0.4) | 23 (0.8) | 57 (1.9) | 78 (2.6) | 2837 (94.3) |
|  | No pregnancy during MS | 1761 | 3 (.2) | 8 (.5) | 16 (.9) | 39 (2.2) | 44 (2.5) | 1651 (93.8) |
|  | Pregnancy during MS | 1248 | 3 (.2) | 0 (0) | 7 (.6) | 18 (1.4) | 34 (2.7) | 1186 (95.0) |
| **Other** | All | 2080 | 17 (0.8) | 46 (2.2) | 39 (1.9) | 26 (1.3) | 22 (1.1) | 1930 (92.8) |
|  | No pregnancy during MS | 1187 | 11 (.9) | 21 (1.8) | 18 (1.5) | 15 (1.3) | 12 (1.0) | 1110 (93.5) |
|  | Pregnancy during MS | 893 | 6 (.7) | 25 (2.8) | 21 (2.4) | 11 (1.2) | 10 (1.1) | 820 (91.8) |

**Table S3*.* Frequency and proportions (%) of all responses to question:** ***Do you receive the support you need from the following professionals?***

| ***Healthcare professional*** | ***All participants***  ***or subgroups*** | ***N,n*** | ***Every week*** | ***Every month*** | ***A few times a year*** |
| --- | --- | --- | --- | --- | --- |
| **Physician at a neurology clinic** | All | 3054 | 186 (6.1) | 518 (17.0) | 2350 (76.9) |
|  | No pregnancy during MS | 1792 | 99 (5.5) | 332 (18.5) | 1361 (75.9) |
|  | Pregnancy during MS | 1262 | 87 (6.9) | 186 (14.7) | 989 (78.4) |
| **Neurological rehabilitation** | All | 2989 | 1906 (63.8) | 490 (16.4) | 593 (19.8) |
|  | No pregnancy during MS | 1757 | 1084 (61.7) | 306 (17.4) | 367 (20.9) |
|  | Pregnancy during MS | 1232 | 822 (66.7) | 184 (14.9) | 226 (18.3) |
| **Physician at another specialist clinic** | All | 3012 | 1950 (64.7) | 245 (8.1) | 817 (27.1) |
|  | No pregnancy during MS | 1763 | 1154 (65.5) | 154 (8.7) | 455 (25.8) |
|  | Pregnancy during MS | 1249 | 796 (63.7) | 91 (7.3) | 362 (29.0) |
| **General practitioner at a primary healthcare center** | All | 3021 | 1508 (49.9) | 303 (10.0) | 1210 (40.1) |
|  | No pregnancy during MS | 1772 | 897 (50.6) | 177 (10.0) | 698 (39.4) |
|  | Pregnancy during MS | 1249 | 611 (48.9) | 126 (10.1) | 512 (41.0) |
| **Nurse** | All | 3023 | 1057 (35.0) | 163 (5.4) | 1803 (59.6) |
|  | No pregnancy during MS | 1768 | 595 (33.7) | 99 (5.6) | 1074 (60.7) |
|  | Pregnancy during MS | 1255 | 462 (36.8) | 64 (5.1) | 729 (58.1) |
| **Medical social worker** | All | 3013 | 2232 (74.1) | 336 (11.2) | 445 (14.8) |
|  | No pregnancy during MS | 1769 | 1288 (72.8) | 209 (11.8) | 272 (15.4) |
|  | Pregnancy during MS | 1244 | 944 (75.9) | 127 (10.2) | 173 (13.9) |
| **Psychologist** | All | 3014 | 2146 (71.2) | 516 (17.1) | 352 (11.7) |
|  | No pregnancy during MS | 1768 | 1261 (71.3) | 321 (18.2) | 186 (10.5) |
|  | Pregnancy during MS | 1246 | 885 (71.0) | 195 (15.7) | 166 (13.3) |
| **Psychotherapist** | All | 3003 | 2511 (83.6) | 276 (9.2) | 216 (7.2) |
|  | No pregnancy during MS | 1761 | 1473 (83.6) | 172 (9.8) | 116 (6.6) |
|  | Pregnancy during MS | 1242 | 1038 (83.6) | 104 (8.4) | 100 (8.1) |
| **Occupational therapist** | All | 3011 | 2235 (74.2) | 301 (10.0) | 475 (15.8) |
|  | No pregnancy during MS | 1763 | 1286 (72.9) | 193 (10.9) | 284 (16.1) |
|  | Pregnancy during MS | 1248 | 949 (76.0) | 108 (8.7) | 191 (15.3) |
| **Physiotherapist** | All | 3007 | 1783 (59.3) | 525 (17.5) | 699 (23.2) |
|  | No pregnancy during MS | 1767 | 1030 (58.3) | 320 (18.1) | 417 (23.6) |
|  | Pregnancy during MS | 1240 | 753 (60.7) | 205 (16.5) | 282 (22.7) |
| **Speech therapist** | All | 3000 | 2762 (92.1) | 46 (1.5) | 192 (6.4) |
|  | No pregnancy during MS | 1762 | 1484 (84.3) | 150 (8.5) | 126 (7.2) |
|  | Pregnancy during MS | 1238 | 1039 (83.9) | 105 (8.5) | 94 (7.6) |
| **Naprapathy / chiropractor** | All | 3006 | 2199 (73.2) | 414 (13.8) | 393 (13.1) |
|  | No pregnancy during MS | 1766 | 1316 (74.5) | 243 (13.8) | 207 (11.7) |
|  | Pregnancy during MS | 1240 | 883 (71.2) | 171 (13.8) | 186 (15.0) |
| **Rehabilitation coordinator** | All | 2998 | 2523 (84.2) | 255 (8.5) | 220 (7.3) |
|  | No pregnancy during MS | 1760 | 1484 (84.3) | 150 (8.5) | 126 (7.2) |
|  | Pregnancy during MS | 1238 | 1039 (83.9) | 105 (8.5) | 94 (7.6) |
| **Dietician** | All | 2950 | 2398 (81.3) | 341 (11.6) | 211 (7.2) |
|  | No pregnancy during MS | 1730 | 1397 (80.8) | 215 (12.4) | 118 (6.8) |
|  | Pregnancy during MS | 1220 | 1001 (82.0) | 126 (10.3) | 93 (7.6) |

*Survey questions related to family planning, MS treatment and its impact*

**Table S4. Frequency and proportions (%) of responses to several questions (Q), considering whether the respondent was pregnant or not while having MS and overall**

|  | No pregnancy during MS  (N=1803) | Pregnancy  during MS  (N=1275) | Overall  (N=3078) | P-value (Chi2 test) |
| --- | --- | --- | --- | --- |
| *Q1: Have you ever talked to your neurologist about aspects of family planning (pregnancy, delivery or postpartum)?* | | |  | **0.015** |
| Yes | 373 (20.7%) | 943 (74%) | 1316 (42.8%) |  |
| No | 511 (28.3%) | 167 (13.1%) | 678 (22.0%) |  |
| Not applicable | 915 (50.7%) | 161 (12.6%) | 1076 (35.0%) |  |
| Missing | 4 (0.2%) | 4 (0.3%) | 8 (0.3%) |  |
| *Q2: From which of the following sources do you mainly get information about MS treatments?* | | | | 0.231 |
| Physician | 1117 (62.0%) | 845 (66.3%) | 1962 (63.7%) |  |
| Other healthcare professionals | 216 (12.0%) | 139 (10.9%) | 355 (11.5%) |  |
| Internet | 365 (20.2%) | 223 (17.5%) | 588 (19.1%) |  |
| Patient association | 30 (1.7%) | 16 (1.3%) | 46 (1.5%) |  |
| Other contacts | 28 (1.6%) | 18 (1.4%) | 46 (1.5%) |  |
| Other | 31 (1.7%) | 21 (1.6%) | 52 (1.7%) |  |
| Missing | 16 (0.9%) | 13 (1.0%) | 29 (0.9%) |  |
| *Q3: Overall, how has your pregnancy/pregnancies affected your MS?* | | | | **-** |
| Positively | - | 296 (23.2%) | - |  |
| Neither positively nor negatively | - | 716 (56.2%) | - |  |
| Negatively | - | 236 (18.5%) | - |  |
| Missing | - | 27 (2.1%) | - |  |
| *Q4: Has your MS treatment affected your choice to breastfeed?* | | | | **-** |
| No | - | 344 (27.0%) | - |  |
| Yes, I have chosen not to breastfeed considering MS treatments | - | 350 (27.5%) | - |  |
| Yes, I have postponed MS treatments to be able to breastfeed | - | 399 (31.3%) | - |  |
| I have not breastfed for other reasons | - | 73 (5.7%) | - |  |
| Missing | - | 109 (8.5%) | - |  |

**Table S5. Frequency and proportions (%) of clinical characteristics by treatment satisfaction response by group and overall**

|  | **No pregnancy during MS** | | | | **Pregnancy during MS** | | | |
| --- | --- | --- | --- | --- | --- | --- | --- | --- |
|  | **Yes** | **Somewhat** | **No** | **Overall** | **Yes** | **Somewhat** | **No** | **Overall** |
|  | N = 1 389 | N = 330 | N = 49 | N = 1 768 | N = 983 | N = 233 | N = 37 | N = 1253 |
| **Latest DMT, n (%)** |  |  |  |  |  |  |  |  |
| No treatment | 54 (3.89%) | 15 (4.55%) | 13 (26.53%) | 82 (4.64%) | 74 (7.53%) | 27 (11.59%) | 13 (35.14%) | 114 (9.10%) |
| Alemtuzumab | 14 (1.01%) | 0 (0.00%) | <5 (2.04%) | 15 (0.85%) | 12 (1.22%) | <5 (1.29%) | 0 (0.00%) | 15 (1.20%) |
| Cladribine | 30 (2.16%) | <5 (0.30%) | 0 (0.00%) | 31 (1.75%) | 17 (1.73%) | <5 (1.29%) | <5 (2.70%) | 21 (1.68%) |
| Dimethyl fumarate | 154 (11.09%) | 54 (16.36%) | 5 (10.20%) | 213 (12.05%) | 88 (8.95%) | 27 (11.59%) | <5 (8.11%) | 118 (9.42%) |
| Fingolimod | 65 (4.68%) | 18 (5.45%) | <5 (2.04%) | 84 (4.75%) | 57 (5.80%) | 16 (6.87%) | <5 (2.70%) | 74 (5.91%) |
| Glatiramer acetate | 29 (2.09%) | 15 (4.55%) | 0 (0.00%) | 44 (2.49%) | 16 (1.63%) | 11 (4.72%) | <5 (2.70%) | 28 (2.23%) |
| HSCT | 23 (1.66%) | 3 (0.91%) | <5 (2.04%) | 27 (1.53%) | 24 (2.44%) | <5 (1.29%) | 0 (0.00%) | 27 (2.15%) |
| Interferon | 56 (4.03%) | 21 (6.36%) | <5 (8.16%) | 81 (4.58%) | 39 (3.97%) | 22 (9.44%) | <5 (5.41%) | 63 (5.03%) |
| Natalizumab | 179 (12.89%) | 38 (11.52%) | <5 (6.12%) | 220 (12.44%) | 153 (15.56%) | 23 (9.87%) | <5 (2.70%) | 177 (14.13%) |
| Ocrelizumab | 15 (1.08%) | <5 (0.30%) | 0 (0.00%) | 16 (0.90%) | 9 (0.92%) | 0 (0.00%) | <5 (2.70%) | 10 (0.80%) |
| Ofatumumab | <5 (0.07%) | 0 (0.00%) | 0 (0.00%) | <5 (0.06%) | 0 (0.00%) | 0 (0.00%) | 0 (0.00%) | 0 (0.00%) |
| Rituximab | 735 (52.92%) | 156 (47.27%) | 20 (40.82%) | 911 (51.53%) | 473 (48.12%) | 95 (40.77%) | 14 (37.84%) | 582 (46.45%) |
| Teriflunomide | 34 (2.45%) | 8 (2.42%) | <5 (2.04%) | 43 (2.43%) | 21 (2.14%) | <5 (1.29%) | 0 (0.00%) | 24 (1.92%) |
| **Latest DMT categories, n (%)** |  |  |  |  |  |  |  |  |
| No treatment | 54 (3.89%) | 15 (4.55%) | 13 (26.53%) | 82 (4.64%) | 74 (7.53%) | 27 (11.59%) | 13 (35.14%) | 114 (9.10%) |
| Low efficacy | 368 (26.49%) | 117 (35.45%) | 11 (22.45%) | 496 (28.05%) | 238 (24.21%) | 82 (35.19%) | 8 (21.62%) | 328 (26.18%) |
| High efficacy | 232 (16.70%) | 42 (12.73%) | 5 (10.20%) | 279 (15.78%) | 198 (20.14%) | 29 (12.45%) | <5 (5.41%) | 229 (18.28%) |
| Rituximab* | 735 (52.92%) | 156 (47.27%) | 20 (40.82%) | 911 (51.53%) | 473 (48.12%) | 95 (40.77%) | 14 (37.84%) | 582 (46.45%) |
| **EDSS, n (%)** |  |  |  |  |  |  |  |  |
| mild | 935 (67.31%) | 186 (56.36%) | 13 (26.53%) | 1 134 (64.14%) | 665 (67.65%) | 124 (53.22%) | 13 (35.14%) | 802 (64.01%) |
| moderate | 135 (9.72%) | 49 (14.85%) | 10 (20.41%) | 194 (10.97%) | 110 (11.19%) | 45 (19.31%) | 8 (21.62%) | 163 (13.01%) |
| severe | 25 (1.80%) | 18 (5.45%) | 11 (22.45%) | 54 (3.05%) | 28 (2.85%) | 17 (7.30%) | 7 (18.92%) | 52 (4.15%) |
| Missing | 294 (21.17%) | 77 (23.33%) | 15 (30.61%) | 386 (21.83%) | 180 (18.31%) | 47 (20.17%) | 9 (24.32%) | 236 (18.83%) |
| **Age groups, n (%)** |  |  |  |  |  |  |  |  |
| 20-29 years | 198 (14.25%) | 31 (9.39%) | <5 (8.16%) | 233 (13.18%) | 43 (4.37%) | 5 (2.15%) | <5 (5.41%) | 50 (3.99%) |
| 30-39 years | 399 (28.73%) | 103 (31.21%) | 12 (24.49%) | 514 (29.07%) | 365 (37.13%) | 83 (35.62%) | 10 (27.03%) | 458 (36.55%) |
| 40-49 years | 642 (46.22%) | 161 (48.79%) | 25 (51.02%) | 828 (46.83%) | 522 (53.10%) | 131 (56.22%) | 23 (62.16%) | 676 (53.95%) |
| 50-59 years | 150 (10.80%) | 35 (10.61%) | 8 (16.33%) | 193 (10.92%) | 53 (5.39%) | 14 (6.01%) | <5 (5.41%) | 69 5.51%) |

* Rituximab is a High-Efficacy DMT. However, due to its extended use in Sweden, counts have been calculated separately.

*Abbreviations*: n, sample size; DMT, disease modifying treatment; EDSS, Expanded disability scale score

*Note*: Cell sizes less than 5 are suppressed as per data access and privacy requirement
