# Supplementary material for: Family planning and multiple sclerosis – Part 1: Patient perspectives on treatment choices in Sweden
Source: Mult Scler J Exp Transl Clin. 2026 Aug 5;12(3):20552173261472661. doi: 10.1177/20552173261472661 (PMC13443254; doi:10.1177/20552173261472661)
Supplement: Supplementary file 2 [file sj-docx-2-mso-10.1177_20552173261472661.docx]

**APPENDIX**

Questions from the survey included in this study:

**Source of information**

“*From which of the following sources do you mainly get information about MS treatments*” with a multiple option response among the following stakeholders:

- Physician
- Other healthcare professionals
- Internet
- Patient association
- other contacts
- Other.

*“How often do you receive care/treatment/support from the following professionals*? - *including both physical visits and, for example, telephone contacts*” with a frequency response to choose among: every week; Every month; A few times a year; The past year; The past 2-3 years; More seldom/ never, and for each of the following professions:

- Physician at a neurology clinic
- Neurological rehabilitation
- Physician at another specialist clinic
- General practitioner at a primary healthcare center
- Nurse
- Medical social worker
- Psychologist
- Psychotherapist
- Occupational therapist
- Physiotherapist
- Speech therapist
- Naprapathy / chiropractor
- Rehabilitation coordinator
- Dietician
- Other: (text)____

“ *Do you receive the support you need from the following professionals?*” (Closed-end option responses: No need; No, would like more; Yes, enough. These responses apply to the same professions mentioned above).

**Pregnancy & breastfeeding**

*“Have you been pregnant during the time you have had MS?* (Closed-end option responses: Yes, once; Yes several times; No. Yes options were collapsed together as “Yes”)

*“Are you pregnant now?”* (closed-end response option: yes/no)

*“Overall, how has your pregnancy/pregnancies affected your MS?”* (response options: Positively, Neither positively nor negatively; Negatively)

*“Has your MS treatment affected your choice to breastfeed?*” *If you have several children, answer according to your most recent child* (Closed-end option responses: No; Yes, I have chosen not to breastfeed considering MS treatments; Yes, I have postponed MS treatments to be able to breastfeed; I have not breastfed for other reasons)

**Treatment**

“Are you satisfied with your treatment?” (response options: Yes; Partly; No)
